# IL-33 Prevents MLD-STZ Induction of Diabetes and Attenuate Insulitis in Prediabetic NOD Mice

**DOI:** 10.3389/fimmu.2018.02646

**Published:** 2018-11-15

**Authors:** Sladjana Pavlovic, Ivica Petrovic, Nemanja Jovicic, Biljana Ljujic, Marina Miletic Kovacevic, Nebojsa Arsenijevic, Miodrag L. Lukic

**Affiliations:** ^1^Faculty of Medical Sciences, Center for Molecular Medicine and Stem Cell Research, University of Kragujevac, Kragujevac, Serbia; ^2^Department of Pathophysiology, Faculty of Medical Sciences, University of Kragujevac, Kragujevac, Serbia; ^3^Department of Histology and Embryology, Faculty of Medical Sciences, University of Kragujevac, Kragujevac, Serbia; ^4^Department of Genetics, Faculty of Medical Sciences, University of Kragujevac, Kragujevac, Serbia

**Keywords:** IL-33, diabetes, C57BL/6 mice, NOD mice, streptozotocin

## Abstract

Type 1 diabetes is an autoimmune disease caused by the immune-mediated destruction of pancreatic β-cells. Prevention of type 1 diabetes requires early intervention in the autoimmune process against beta-cells of the pancreatic islets of Langerhans, which is believed to result from disordered immunoregulation. CD4^+^Foxp3^+^ regulatory T cells (Tregs) participate as one of the most important cell types in limiting the autoimmune process. The aim of this study was to investigate the effect of exogenous IL-33 in multiple low dose streptozotocin (MLD-STZ) induced diabetes and to delineate its role in the induction of protective Tregs in an autoimmune attack. C57BL/6 mice were treated i. p. with five doses of 40 mg/kg STZ and 0.4 μg rIL-33 four times, starting from day 0, 6, or 12 every second day from the day of disease induction. 16 weeks old NOD mice were treated with 6 injections of 0.4 μg/mouse IL-33 (every second day). Glycemia and glycosuria were measured and histological parameters in pancreatic islets were evaluated at the end of experiments. Cellular make up of the pancreatic lymph nodes and islets were evaluated by flow cytometry. IL-33 given simultaneously with the application of STZ completely prevented the development of hyperglycemia, glycosuria and profoundly attenuated mononuclear cell infiltration. IL-33 treatment was accompanied by higher number of IL-13 and IL-5 producing CD4^+^ T cells and increased presence of ST2^+^Foxp3^+^ regulatory T cells in pancreatic lymph nodes and islets. Elimination of Tregs abrogated protective effect of IL-33. We provide evidence that exogenous IL-33 completely prevents the development of T cell mediated inflammation in pancreatic islets and consecutive development of diabetes in C57BL/6 mice by facilitating the induction Treg cells. To extend this finding for possible relevance in spontaneous diabetes, we showed that IL-33 attenuate insulitis in prediabetic NOD mice.

## Introduction

Diabetes mellitus type 1 is a chronic inflammatory disease characterized by the progressive destruction of pancreatic β-cells of Langerhans islets caused by autoimmune processes (1–3). The development of these autoimmune processes is thought to be the result of disorders of immunoregulation (3). This failure allows Th1/Th17 lymphocytes to trigger a cascade of immune/inflammatory processes in the pancretic islets causing β cell destruction. Both the numerical and functional equilibrium between effector and regulatory T lymphocytes in pancreatic infiltrates determine the extent of destruction of β cells (4–6).

IL-33 is a member of the IL-1 cytokine family (7). Receptor for IL-33 is ST2 (IL-33R) molecule that is constitutively expressed on various cells including subpopulation of Foxp3 regulatory cells Th2 lymphocytes, innate lymphoid cells (ILC2), mast cells, basophiles, and eosinophils (8–10). IL-33/ST2 axis is important in several inflammatory diseases (11, 12).

The idea behind this work stems from our previous data on the role of IL-33/ST2 axis in the models of the two Th1 mediated diseases: MLD-STZ and experimental allergic encephalomyelitis (EAE) (5, 13–15). Deletion of ST2 abrogates resistance to EAE in BALB/C mice by enhancing polarization of antigen presenting cells to inflammatory phenotypes and enhancing IL-17 and IFN-γ production. In MLD-STZ diabetes model we showed that resistance to disease in BALB/C mice depends partially on CD4^+^CD25^+^Foxp3^+^ cells (5, 15).

Further, Yuan et al. (16) recently showed that in prediabetic NOD mice a combination of CD122 and IL-33 promotes Tregs abundance and function in pancreatic islets. Finally Ryba-Stanislawowska et al. (17) reported that *in vitro* IL-33 treatment of Tregs derived from patients with type 1 diabetes resulted in quantitative and qualitative enhancement of their suppressive activity.

Siede et al. (18) have reported that IL-33 receptor expressing Treg cells acquire capacity to produce IL-5 and IL-13 and suppress T effectors cells by producing IL-10. Taken together these data suggested that *in vivo* treatment of IL-33 may have beneficial effects in MLD-STZ diabetes by promoting Tregs and in particular ST2^+^ Tregs producing IL-10 and possibly IL-5 and/or IL-13.

MLD-STZ induced diabetes appears to be an experimental model for studying T cell-dependent inflammatory pathology in the islets (19). We used this model to investigate the immunomodulatory capacity of IL-33 and to delineate the mechanisms influencing effectors immune cell functions. Our study has shown that IL-33 prevents MLD-STZ diabetes induction if given at the time of disease induction. If given 6 and 12 days after the disease induction IL-33 can still significantly attenuate development of hyperglycemia. Finally, in order to show relevance of our findings for the development of “spontaneous” diabetes, we looked at the possibility that exogenous IL-33 alter the onset of insulitis in prediabetic NOD mice. IL-33 treated NOD mice showed significantly lower mononuclear cells infiltration but higher percentage and number of CD4^+^IL-5^+^, CD4^+^IL-13^+^, and CD4^+^Foxp3^+^ cells expression in the islets.

This beneficial effect appears to be mainly due to the ability of IL-33 to enhance induction of regulatory CD4^+^Foxp3^+^ ST2^+^ T cells.

## Materials and methods

### Experimental animals

C57BL/6 mice male 8–10 week old, housed under conventional conditions and allowed laboratory chow and water *ad libitum*, were used in the experiments. Within each experiment, animals were matched by age and weight (18–24 g) and randomly divided into groups of 7–8 to receive different treatments. Breeding pairs of NOD mice were purchased from Charles River Laboratories SRL (Calco, Italy) and maintained in specific pathogen free facilities. Female NOD mice at the 16 week of age were used in the experiment. In our animal facilities only 25–35% of NOD mice develop diabetes typically at the 24th week of age. In attempt to standardize the evaluation of IL-33 in these mice we used group of 20 female mice at the age of 16 weeks and 10 of them were treated with IL-33.

All animal procedures were approved by the Ethics Committee for Animal studies of the Faculty of Medical Science, University in Kragujevac.

### Diabetes induction

Diabetes was induced by MLD-STZ, as described earlier (20). Briefly, animals received intraperitoneally (i. p.) 40 mg/kg (b.w.) STZ (Sigma-Aldrich, St Louis, MO) dissolved in citrate buffer on pH 4.5 for 5 consecutive days. The daily dose of STZ is determined by the daily weight measurement of each mouse immediately prior to the injection of the substance.

### Diabetes evaluation

Clinical diabetes was defined by hyperglycaemia (blood glucose levels > 10.3 mmol/l) and glycosuria (urine glucose levels > 7.1 mmol/l or 128 mg/100 ml) in fasted animals. Blood glucose levels and urine glucose were measured three times per week. Samples were taken from the tail tip after starvation for 4 h. Blood glucose levels (mmol/l) were determined using the Accu-Chek Performa glucometer (Roshe Diagnostics, Mannheim, Germany) and urine glucose was analyzed by Uriscan 2 test strip (YD diagnostics, Korea).

### Glucose tolerance test (GTT)

GTT analyses were performed both in C57BL/6 and NOD mice at the end of experiment. To this end food was withheld 16 h before testing. Animals were weighed and injected with 2 g/kg of glucose (i.p.). Glucose concentrations were measured before and at 0, 15, 30, 60, and 120 min after glucose injection.

### IL-33 application

C57BL/6 mice received exogenous mouse IL-33 (0.4 μg/injection; eBioscience) intraperitoneally, according to the following scheme: I group: days 0, 2, 4, and 6; II group: days 6, 8, 10, and 12; III group: days 12, 14, 16, and 18. Control animals were treated with intraperitoneally PBS + citrate buffer (CB) solution or IL-33 + citrate buffer solution at the same time interval.

The effects of IL-33 on development of periinsulitis and insulitis in prediabetic NOD mice were evaluated. Sixteen weeks old animals that were all normoglycemic, free of glycosuria and with normal GTT test were treated with 6 injections of 0.4 μg/mouse IL-33 (every second day) and sacrificed for histological analysis at 18th week of age.

### Cyclophosphamide (CY) administration

CY was administered at a dose 200 mg/kg of body weight twice on 5 and 7th day of the treatment. The first CY injection was given ~8 h after the last administration of STZ as a precaution against a possible interaction of CY with STZ (21).

### Histological examination of pancreata

Pancreata of all groups were excised and placed in 10% buffered formaldehyde fixative solution overnight at room temperature. Pancreatic tissue paraffin sections were stained with hematoxylin-eosin were used for the analysis of lymphocytic infiltrates in the Langerhans pancreatic islets by light microscope (BX51; Olympus, Japan) using a magnification lens of 40 × (14). Histological analysis of the distribution of inflammatory cell infiltrate in pancreatic islets was performed in blinded fashion by two independent observers (MM and SP). Insulitis was graded and a mean insulitis score was calculated as described previously (22).

### Isolation of lymphoid cells for phenotypic assessment

Pancreatic draining lymph nodes were removed, and single-cell suspensions were obtained by mechanical disruption. After removing pancreatic lymph nodes, pancreas was processed through three steps: *in situ* perfusion with collagenase, pancreatic digestion, and isolation of the islet. The cells were separated according to the protocol as describe elsewhere (23) and analyzed by flow cytofluorimetry. Data was shown as percentage of mononuclear cells and absolute number of cells per islets from one pancreas.

### Flow cytometric analysis

Cells suspensions were prepared from lymph nodes and pancreatic islets. Single-cell suspensions were labeled with fluorochrome-conjugated monoclonal antibodies: anti-mouse CD3, CD4, CD8, ST2, and CXCR3 (BD Biosciences), CD11c and CD11b antibodies (BioLegend, San Diego, CA) or with isotype-matched control and analyzed on a FACSCalibur (BD) using CELLQUEST software (BD). The intracellular staining was performed with lymph node cells incubated for 6 h in the presence of Phorbol 12-myristate13-acetate (50 ng/ml) (Sigma, USA), Ionomycin (Sigma, USA) (500 ng/ml), and GolgyStop (BD Pharmingen) at 37°C, 5% CO2, stained with anti-CD4 monoclonal antibodies or appropriate isotype controls, fixed and permeabilized with a Cytofix/Cytoperm solution. Intracellular staining was performed using monoclonal antibodies: IFN-γ, IL-17, IL-10, IL-5, IL-13, IL-2, and Foxp3 (BD Biosciences) or appropriate negative controls. Cells were analyzed with the FACSCalibur Flow Cytometer (BD Biosciences), and analysis was conducted with FlowJo (Tree Star).

### Statistical analysis

All variables were continuous and values were described by the means ± SEM. In order to determine differences in the mean values of continuous variables with a normal distribution of values, parametric Student's *t*-test was used, and its non-parametric alternative Mann-Whitney test if data did not follow a normal distribution. All data were analyzed using the statistical program SPSS version 20 (SPSS Inc., Chicago, IL) where *p*-value < 0.05 was considered statistically significant.

## Results

### IL-33 treatment prevents diabetes induction in C57BL/6 mice as evaluated by glycemia, glucose tolerance test, glycosuria, and islet infiltration

**Figure 1:**
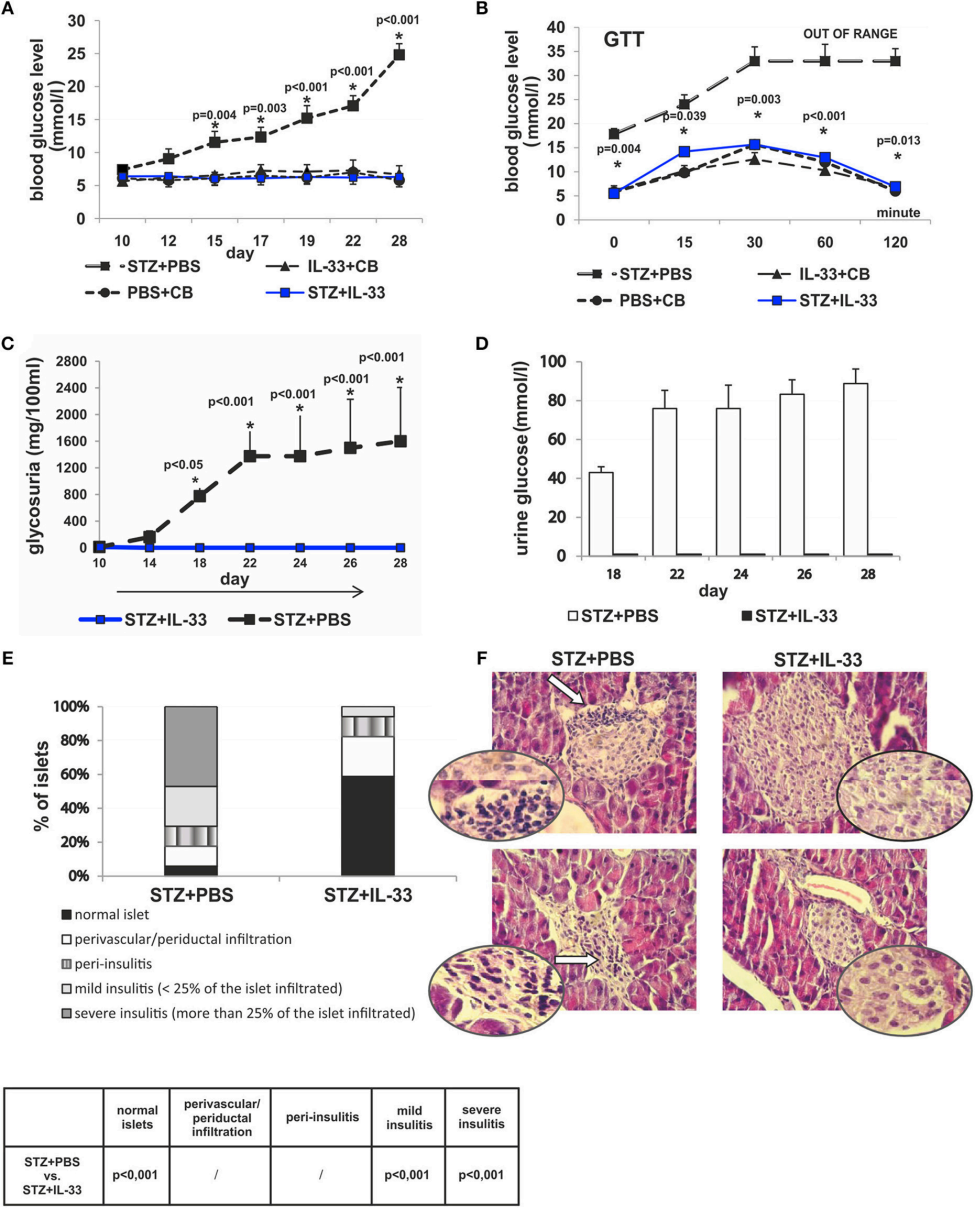
Concomitant treatment with recombinant IL-33 completely abrogates induction of diabetes by MLD-STZ. Effect of four injections of 0.4 μg/mouse IL-33 on glycemia **(A)**, GTT test **(B)**, and glycosuria **(C,D)**. Histology of the islets showed highly significant (*p* < 0.001) decrease of mononuclear cells influx in IL-33 treated group compared to control group **(E,F)**. An analysis of lymphocytic infiltrates in Langerhans's pancreatic islands was performed by light microscope using a magnifying lens of 40 X.

Present study was undertaken to analyze the effect of exogenous IL-33 on the onset and the development of type 1 diabetes as evaluated by glycemia, glucose tolerance test, glycosuria, and islet infiltration. C57BL/6 mice were injected with four injections of IL-33 or PBS starting from the day 0 as described in material and methods section (I group). As shown in Figure 1, exogenous IL-33 showed strong suppressive effects on diabetes induction and no biochemical parameters of the disease onset were noticed. Significant difference in values of glycemia was observed from the day 15 and remained until the end of the experiment (Figure 1A).

We examined blood sugar values of the experimental mice after glucose loading using a glucose tolerance test. All measurements were conducted within 120 min in line with the schedule: 0, 15, 30, 60, and 120. Assessing the differences between the group that received IL-33 from the day 0 and the control group, we found significant differences in all five time points. Significances (*p*-values) according to the schedule of measurements were as follows: 0.004, 0.039, 0.003, < 0.001, and 0.013, respectively (Figure 1B). Control animals did not develop hyperglycemia by the end of the experiment (Figures 1A,B).

We also evaluated the onset and the differences in levels of glycosuria between the observed groups of animals. Measurements were performed on days 10, 14, 18, 22, 24, 26, and 28. First two measurements (days 10 and 14) did not detect glucose in animal's urine in either group. Glycosuria occurred on the day 18 in mice treated with STZ only. Complete prevention of glycosuria was achieved by administration IL-33 (Figures 1C,D).

The degree of insulitis was graded according to Hall et al. (22) and Pejnovic et al. (24): normal islet, score 1; perivascular/periductal infiltration, score 2; peri-insulitis, score 3; mild insulitis (< 25% of the islet infiltrated), score 4; and severe insulitis (more than 25% of the islet infiltrated), score 5. The histological examination of the pancreata revealed differences in the intensity of mononuclear cells infiltration in the islets. No islets with severe insulitis were detected in the group of mice treated with IL-33, while the control group of mice had 47.06% of islets with severe insulitis (*p* < 0.001). The percentage of total intact islets was 58.82% in IL-33 treated mice compared to control group of mice with 5.8% of healthy islets (*p* < 0.001) (Figures 1E,F).

### Exogenous IL-33 particularly enhances the number of CD^4+^Foxp^3+^ cells in pancreatic lymph nodes in C57BL/6 mice

**Figure 2:**
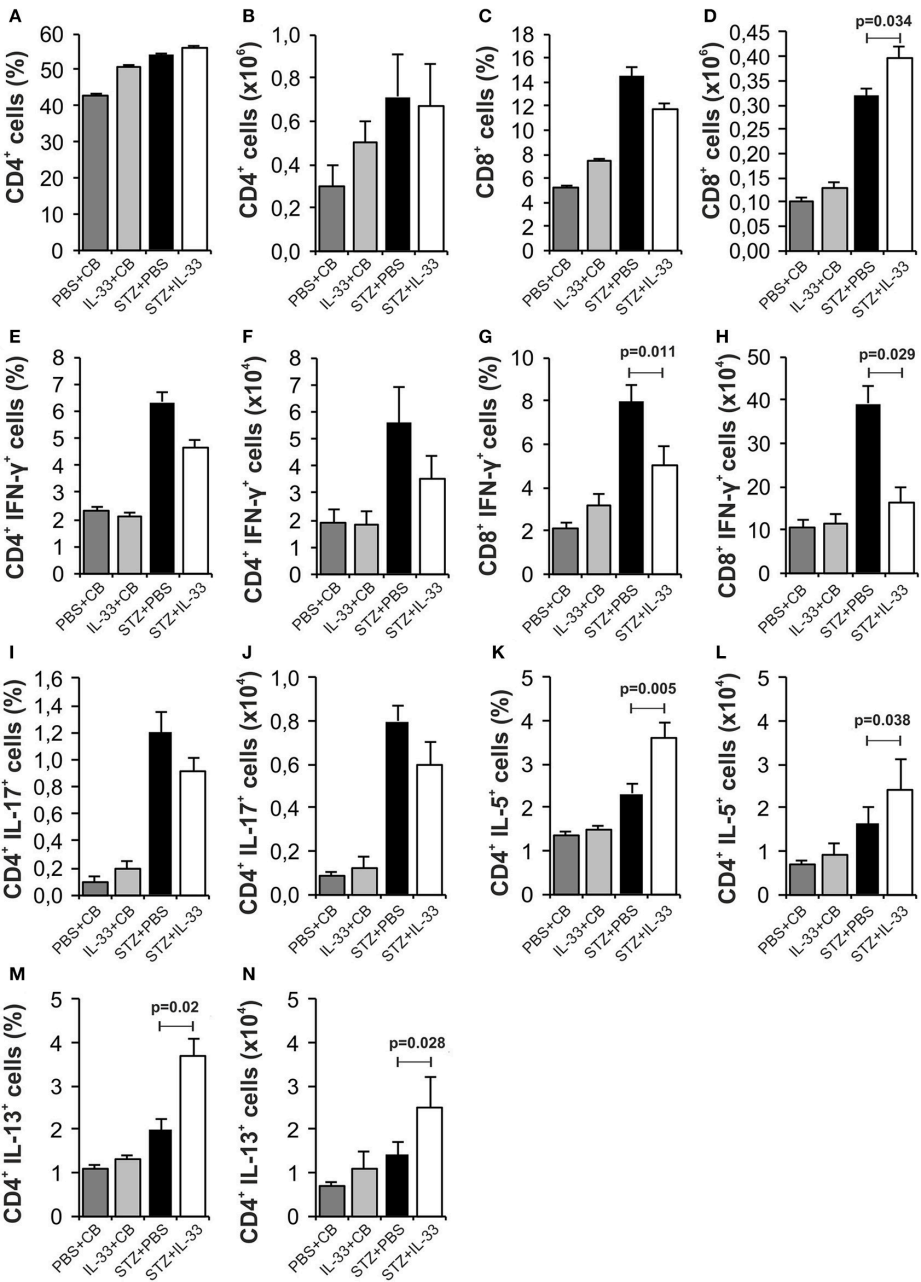
IL-33 attenuates inflammatory cells and increases IL-5 and IL-13 producing CD4^+^ cells. The percentage and total number of CD4^+^** (A,B)**, CD8^+^** (C,D)**, CD4^+^IFN-γ^+^ (**E,F)**, CD8^+^IFN-γ^+^** (G,H)**, CD4^+^IL-17^+^** (I,J)**, CD4^+^IL-5^+^** (K,L)**, CD4^+^IL-13^+^** (M,N)** were examined. The animals were treated with 0.4 μg/injection IL-33 together with MLD-STZ (i.p. 40 mg/kg for 5 consecutive days) or of an equimolar dose of PBS or 0.4 μg/injection IL-33 together with citrate buffer. Cells were obtained from pancreatic lymph nodes on day 28 after diabetes induction. Data from two individual experiments with at least 8 mice per group are shown as mean ± SEM; by paired *t*-test when compared with values obtained with phosphate-buffered saline.

IL-33 did not cause significant differences in the total number and percentage of CD4^+^ (Figures 2A,B) and CD8^+^ lymphocytes (Figures 2C,D) in pancreatic lymph nodes of examined animals. There were no significant differences in percentage and total number of IFN-γ producing CD4^+^ T lymphocytes between the group that were treated with IL-33 and the group that were treated with STZ only (Figures 2E,F). IL-33 treatment significantly reduced the percentage of CD8 lymphocytes producing IFN-γ (*p* = 0.011) (Figure 2G). Likewise, the absolute number of CD8^+^IFN-γ^+^ cells was also significantly lower in the group of mice treated with IL-33, compared to the control group (*p* = 0.029) (Figure 2H). There was no significant difference in the percentage and number of IL-17 producing CD4^+^cells (Figures 2I,J). IL-33 treatment significantly increased the percentage (*p* = 0.005) and the total number (*p* = 0.038) of CD4^+^IL-5^+^ cells (Figures 2K,L) and the percentage and total number of CD4^+^IL-13^+^ cells (*p* = 0.02, *p* = 0.028, respectively) (Figures 2M,N).

**Figure 3:**
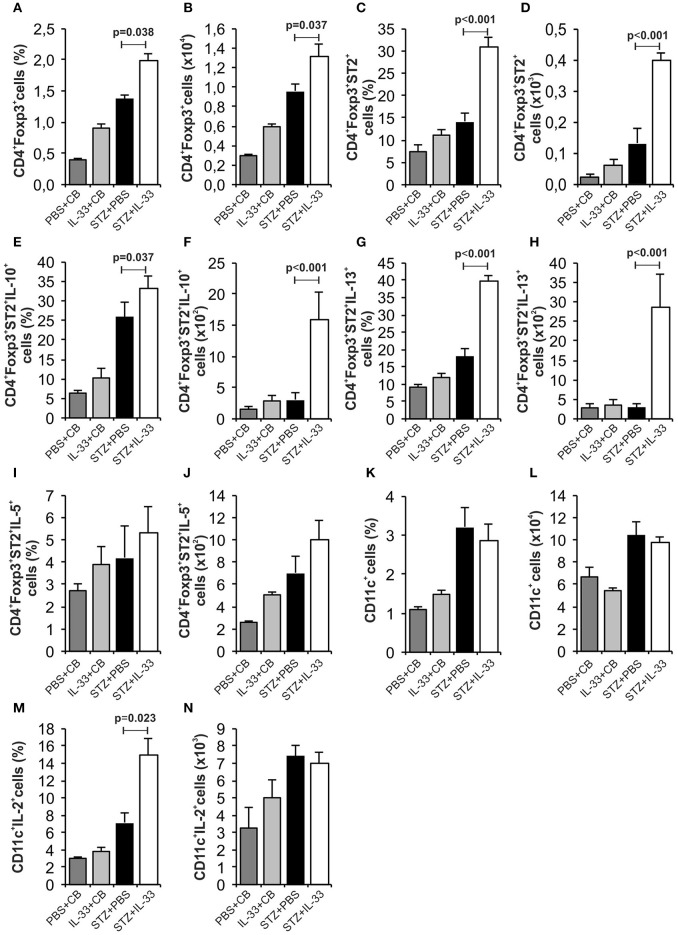
Exogenous IL-33 significantly increases the percentage and total number of regulatory T cells (Foxp3^+^ST2^+^IL-10^+^) in the pancreatic lymph node. The percentage and total number of CD4^+^Foxp3^+^** (A,B)**, CD4^+^Foxp3^+^ST2^+^** (C,D)**, CD4^+^Foxp3^+^ST2^+^IL-10^+^** (E,F)**, CD4^+^Foxp3^+^ST2^+^IL-13^+^** (G,H)**, CD4^+^Foxp3^+^ST2^+^IL-5^+^** (I,J)**, CD11c^+^** (K,L)**, CD11c^+^IL-2^+^** (M,N)** were examined. The animals were treated with 0.4 μg/injection IL-33 together with MLD-STZ (i.p. 40 mg/kg for 5 consecutive days) or of an equimolar dose of PBS or with 0.4 μg/injection IL-33 together with citrate buffer. Cells were obtained from pancreatic lymph nodes on day 28 after diabetes induction. Data from two individual experiments with at least 8 mice per group are shown as mean ± SEM; by paired *t*-test when compared with values obtained with phosphate-buffered saline.

We noticed a significant increase in the percentage (*p* = 0.038) and number (*p* = 0.037) of regulatory CD4^+^Foxp3^+^ cells in the group treated with IL-33 (Figures 3A,B). Further, the IL-33 treatment led to highly significant increase of the percentage and the number of Treg cells expressing ST2 molecule (*p* < 0.001) (Figures 3C,D). Exogenous IL-33 significantly increased percentage and number of CD4^+^Foxp3^+^ST2^+^IL-10^+^ (*p* = 0.037, *p* < 0.001, respectively) (Figures 3E,F), CD4^+^Foxp3^+^ST2^+^IL-13^+^ (*p* < 0.001, *p* < 0.001, respectively) (Figures 3G,H) and percentage of CD4^+^Foxp3^+^ST2^+^IL-5^+^ (*p* < 0.001) (Figure 3J) cells in pancreatic lymph nodes. However, IL-33 treatment did not increase the percentage and number of CD11c^+^ cells (Figures 3K,L), but significantly increased percentage of CD11c^+^ population that produces IL-2 which is important for the survival of Treg cells (*p* = 0.023; Figure 3M). Dot plots relevant for Figures 2, 3 are given in Supplementary Figure S1.

### IL-33 treatment leads to significant decrease in CXCR^3+^ diabetogenic cells in the islets

**Figure 4:**
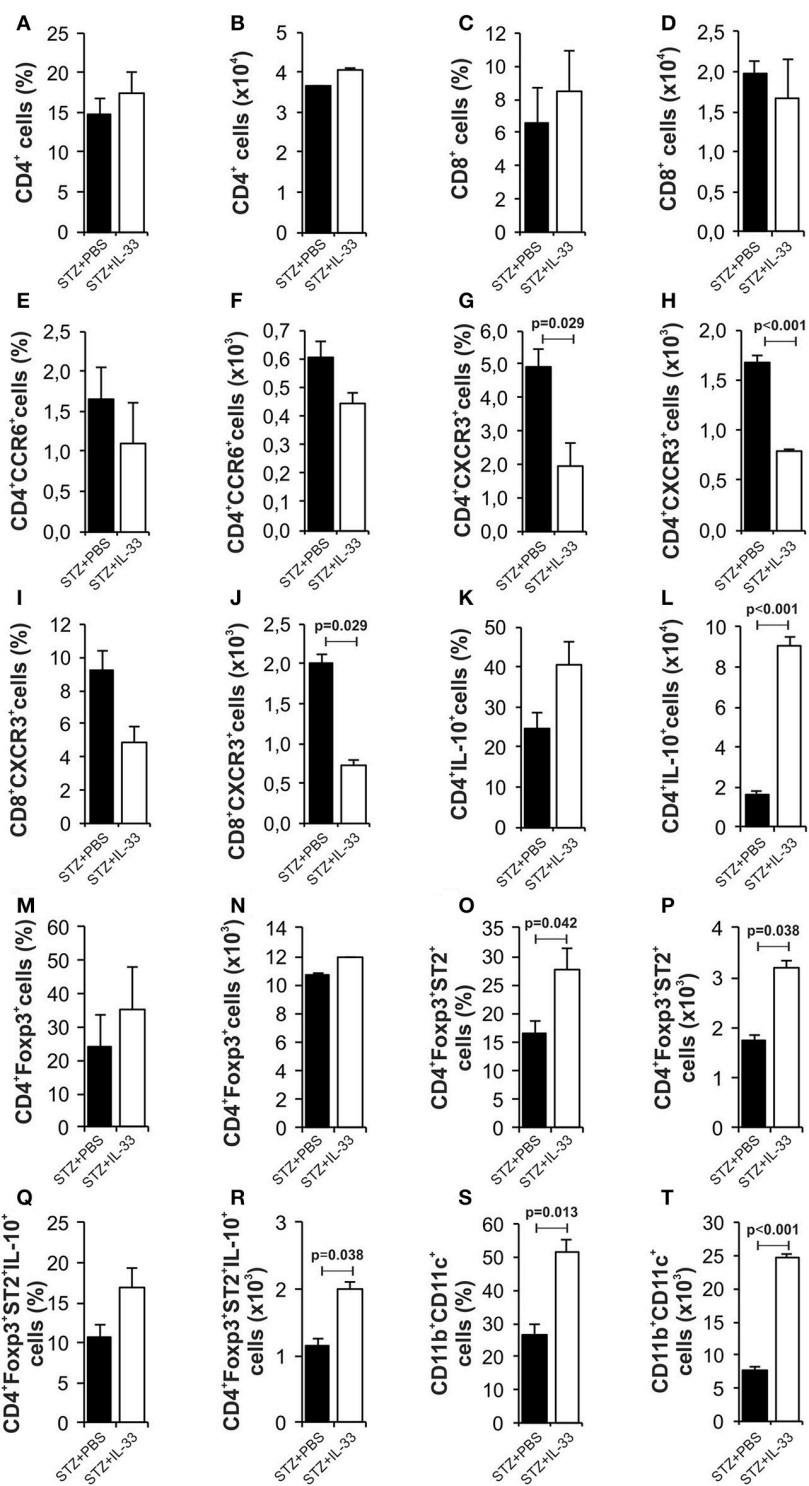
IL-33 attenuates influx of inflammatory cells and promotes regulatory cells infiltration in the pancreatic islets. The percentage and total number of CD4^+^** (A,B), CD4**^**+**^**CCR6**^**+**^** (C,D)**, CD4^+^CXCR3^+^** (E,F)**, CD4^+^IL-10^+^** (G,H)**, CD8^+^** (I,J)**, CD8^+^CXCR3^+^** (K,L)**, CD4^+^Foxp3^+^** (M,N)**, CD4^+^Foxp3^+^ST2^+^** (O,P)**, CD4^+^Foxp3^+^ST2^+^IL-10^+^** (Q,R)**, CD11b^+^CD11c^+^** (S,T)** were examined. IL-33 treatment significant decreases effector CD4^+^CXCR3^+^ and CD8^+^CXCR3^+^ cells and increases percentage of regulatory cells, myeloid dendritic cells. The animals were treated with 0.4 μg/injection IL-33 together with MLD-STZ (i.p. 40 mg/kg for 5 consecutive days) or of an equimolar dose of PBS or with 0.4 μg/injection IL-33 together with citrate buffer. Cells were obtained from pancreatic islets on day 28 after diabetes induction. Data from two individual experiments with at least 8 mice per group are shown as mean ± SEM; by paired *t*-test when compared with values obtained with phosphate-buffered saline.

We also investigated mononuclear infiltrate in pancreatic islets at the end of the experiments (Figure 4). There was no difference in the percentage and number of CD4^+^ cells (Figures 4A,B) and CD8^+^ cells (Figures 4C,D). Results showed significantly decreased percentage (*p* = 0.029) and number (*p* < 0.001) of effectors CD4^+^CXCR3^+^ cells in the group of mice treated with IL-33 (Figures 4G,H). Similarly, we noticed lower percentage of CD8^+^CXCR3^+^ cells (Figures 4I,J) and higher percentage of CD4^+^IL-10^+^ cells (Figures 4K,L) but differences did not reach statistical significance. The treatment with IL-33 induced higher percentage (*p* = 0.042) and total number (*p* = 0.038) of CD4^+^Foxp3^+^ST2^+^ (Figures 4O,P) in mononuclear cell population in the islets. Total number of CD4^+^Foxp3^+^ST2^+^IL-10^+^ cell was also increased (*p* = 0.038) (Figures 4Q,R). Furthermore, we have shown that exogenous IL-33 increased the percentage and the total number of tolerogenic dendritic CD11b^+^CD11c^+^ cells (*p* = 0.013, *p* < 0.001, respectively) (Figures 4S,T) in mononuclear cell population in the islets. Dot plots relevant for Figure 4 are given in Supplementary Figure S2.

### Low dose of cyclophosphamide (CY) affects regulatory cells and attenuates protective effect of IL-33 in autoimmunity including type 1 diabetes

**Figure 5:**
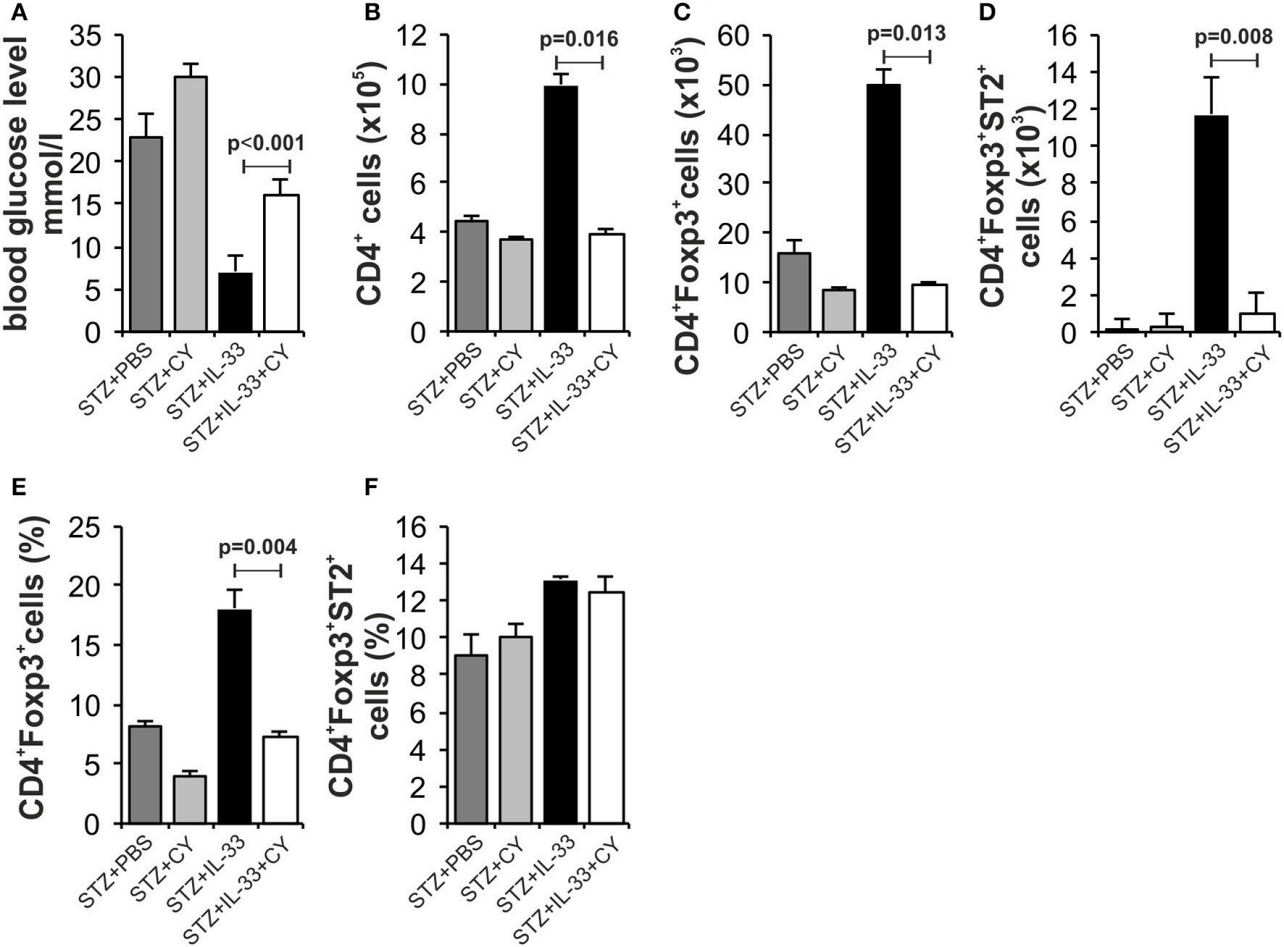
Low dose of cyclophosphamide (CY) affects regulatory cells and attenuates protective effect of IL-33. In order to confirm our hypothesis that the effect of Il-33 administration leads to activation and an increase in the number of regulatory T cells, we added a group that, in addition to MLD STZ and IL-33, also received a low dose of CY (2 × 100 mg/kg) that eliminates regulatory T cells (21). Effect of four injections of 0.4 μg/mouse IL-33, MLD STZ and CY on glycemia **(A)**. The number of CD4^+^** (B)**, CD4^+^Foxp3^+^** (C)**, CD4^+^Foxp3^+^ST2^+^** (D)** in pancreatic lymph nodes and percentage of CD4^+^Foxp3^+^** (E)**, CD4^+^Foxp3^+^ST2^+^** (F)** in pancreatic islets. The animals were treated with MLD-STZ (i.p. 40 mg/kg for 5 consecutive days) with PBS or 0.4 μg/injection of IL-33 or CY or both. Cells were obtained from pancreatic islets and pancreatic lymph nodes on day 28 after diabetes induction. Data from one experiment with at least 8 mice per group are shown as mean ± SEM; by paired *t*-test.

We and others have shown previously that in mice (15) and rats (25) low dose of CY enhances diabetes induction by affecting regulatory cells. Therefore, we tested by day 18 whether protective effect of IL-33 will be affected by pretreatment with CY. There was significant difference in the streptozotocin induced level of glycemia between the groups treated with IL-33 + CY and IL-33 only (*p* < 0.001). Animals treated with STZ + PBS showed high glycemia (23 ± 2.16) vs. animals treated with IL-33+CB or PBS+CB (glycemia 6.8 ± 0.36; 7.0 ± 0.28) which did not develop disease (Figure 5A).

Phenotyping of the cells in pancreatic lymph nodes at the end point of the experiment confirmed the low total number of CD4^+^Foxp3^+^ positive cells (*p* = 0.013) (Figure 5C) as well as CD4^+^Foxp3^+^ST2^+^ cells (*p* = 0.008) (Figure 5D) after CY treatment that correlated with higher glycemia. The percentage of CD4^+^Foxp3^+^ positive cells was also increased in pancreatic islets (*p* = 0.004) (Figure 5E). These data suggest that CD4^+^Foxp3^+^ (ST2^+^) cells are major downregulatory cells induced by IL-33 in MLD-STZ diabetes.

### IL-33 downregulates diabetes if given after the onset of disease

**Figure 6:**
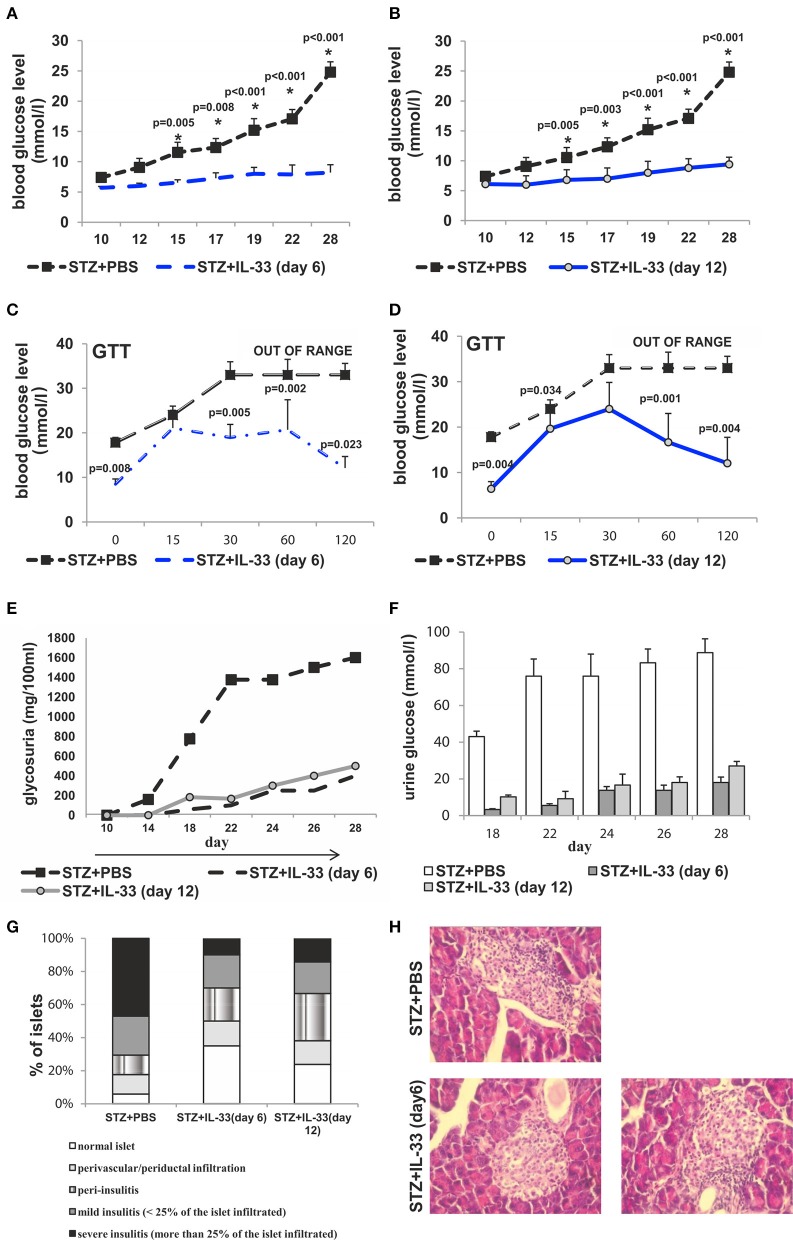
IL-33 given 6 and 12 days after diabetes induction partially attenuates clinical signs and influx of inflammatory cells in the islets. Effect of four injections of 0.4 μg/mouse IL-33 on glycemia **(A,B)**, GTT test **(C,D)** and glycosuria **(E,F)** Histology of the islet showed significantly (*p* < 0.001) decreased influx of mononuclear cells in IL-33 treated group in comparison with control group **(G,H)**. An analysis of lymphocytic infiltrates in Langerhans's pancreatic islands was performed by light microscope using a magnifying lens of 40 X.

Exogenous IL-33 treatment, applied after MLD-STZ, had also antidiabetogenic effects in mice received IL-33 from the day 6 and 12, respectively (Figure 6). In the groups of mice that received IL-33 from day 6, two out of 7 animals and from day 12 three out of 7 developed hyperglycemia presenting the partial effect of IL-33 if given after the onset of disease. There was significant difference in glycemia level between IL-33 treated and control animals (Figures 6A,B).

GTT results were significantly different when comparing the group that received IL-33 from the day 6 to control with the exception of GTT measurement in minute 15 showing similar results within the groups. Quantifications of GTT in remaining four-time intervals were significantly different with *p*-values in minute 0, 0.008; min 30, 0.005; min 60, 0.002; min 120, 0.023 (Figure 6C). Comparing group that received IL-33 from the day 12 with control, the only result with similar GTT levels was evaluated in min 30. Remaining four of scheduled measurements revealed significant differences with *p*-values of 0.004, 0.034, 0.001, and 0.004, respectively (Figure 6D).

Initiated on the day 6 and 12, IL-33 caused a development of a low level of glycosuria. Those low levels were significantly different (*p* = 0.011 from day 18) when IL-33 was initiated on the day 6 and remained different until the end of the experiment on the day 28 (*p* = 0.044) when compared with MLD-STZ treated mice (Figures 6E,F).

### IL-33 treatment attenuates insulitis in prediabetic NOD mice

**Figure 7:**
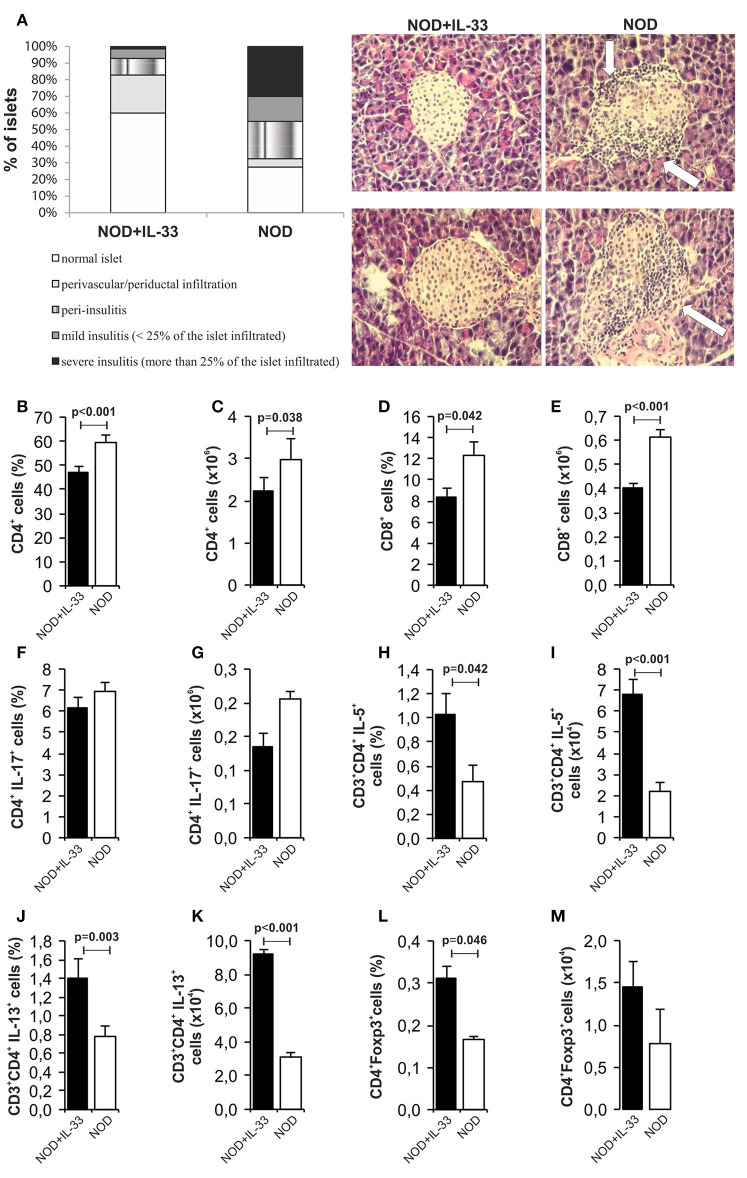
IL-33 decrease insulitis and change composition of mononuclear islet infiltration in prediabetic NOD mice. 16 weeks old animals that were all normoglycemic, free of glycosuria and with normal GTT test were treated with 6 injections of 0,4 μg/mouse IL-33 (every second day) and sacrificed for histological analysis at 18th week of age **(A)**. The percentage and number of CD4^+^** (B,C)**, CD8^+^** (D,E)**, CD4^+^IL-17^+^** (F,G)**, CD3^+^CD4^+^IL-5^+^** (H,I)**, CD3^+^CD4^+^IL-13^+^** (J,K)**, CD4^+^Foxp3^+^** (L,M)** was examined by flow cytometric analysis. IL-33 treatment significant increase percentage of CD3^+^CD4^+^IL-5^+^** (H,I)**, CD3^+^CD4^+^IL-13^+^** (J,K)** and regulatory CD4^+^Foxp3^+^** (L,M)** cells. Data from one experiment with 10 mice per group are shown as mean ± SEM; by paired *t*-test when compared with values obtained with phosphate-buffered saline.

In our further analysis, we wanted to examine the effect of exogenous IL-33 on the development of mononuclear infiltrates in spontaneous diabetes NOD mice. Our results clearly showed a significant difference in the infiltration of pancreatic islets among the mice received IL-33 and untreated NOD mice. We evaluated 70 islets and found 60% intact islets in the IL-33 treated group of mice and only 27.5% in the untreated group. Furthermore, in untreated group of mice, 45% of the islets were affected by severe or mild insulitis (Figure 7A). Exogenous IL-33 significantly decreased percentage (*p* < 0.001) and number (*p* = 0.038) of CD4^+^ (Figures 7B,C) cells in NOD mice. Significantly decreased percentage (*p* = 0.042) and number of CD8^+^ T cells (*p* < 0.001) (Figures 7D,E) was also noticed after IL-33 treatment in NOD mice. The treatment with IL-33 induced higher percentage and number of of CD4^+^IL-5^+^ (*p* = 0.042, *p* < 0.001, respectively) (Figures 7H,I) and CD4^+^IL-13^+^ (*p* = 0.003; *p* < 0.001, respectively) (Figures 7J,K). Percentage of CD4^+^Foxp3^+^ T cells was significantly increased after IL-33 treatment (*p* = 0.046) (Figure 7L) in pancreatic lymph nodes. There was no significant difference between groups in percentage and number of CD4^+^IL-17^+^ cells (Figures 7F,G). Dot plots relevant for Figure 7 are give in Supplementary Figure S3.

## Discussion

In this paper we describe the experiments showing that exogenous IL-33 may prevent development of MLD-STZ diabetes in C57BL/6 mice and significantly attenuate development of insulitis in prediabetic NOD mice. These effects were accompanied by alteration of inflammatory cellular make up in the draining pancreatic lymph nodes as well as in the islets of the pancreas.

There is growing evidence suggesting that IL-33/ST2 axis plays an important role in chronic in?ammatory and autoimmune diseases (26); type 2 diabetes (27), in?ammatory bowel disease (28), cardiac disease (29), graft-vs.-host disease (30) and small bowel transplant rejection (31). Lack of IL-33/ST2 signaling enhances acute hepatitis (32) and EAE (14).

In this paper we add the evidence that not only genetic deletion of IL-33 receptor (ST2) enhance T cell mediated autoimmune inflammatory diseases but also the exogenous IL-33 has shown powerful preventive effect in a model of type 1 diabetes (Figure 1). Moreover, an attempt to therapeutically apply IL-33, 6 and 12 days after diabetes induction had significant effects on the development of clinical and laboratory signs of the disease (Figure 6). This protective effect was also confirmed by the GTT test. Furthermore, IL-33 significantly reduced mononuclear infiltration in the pancreatic islets and we did not notice infiltration in vast majority of IL-33 treated mice. Same animals developed only mild periductal infiltrations (Figure 1).

Interestingly Oboki et al. (33) did not find the differences in susceptibility to MLD-STZ diabetes between IL-33^−/−^ and “wild type mice.” Although the reason for the discrepancy with our results is not clear, it may be assumed that the lack of endogenous IL-33 does not alter other regulatory mechanisms such as ST2 negative T regulatory cells observed in our previous experiments (15).

It was shown that IL-33-activated dendritic cells (DCs) do not produce IL-12, while LPS-activated DCs produce abundant IL-12. It is believed that the pathway IL-33/ST2 may counteract the LPS/TLR4 pathway and thus may control the production of ?h1 cytokines (34). Therefore, it was important to analyse cellular events in the draining pancreatic lymph node and the islets (Figures 2, 4). Application of IL-33 modulates the Th1-Th2 balance and promotes Th2 cells only when given in early phase of the immune response during parasitic infection (35). Similarly, in our investigation the treatment with exogenous IL-33 led to an increase of total number and percentage CD4^+^IL-5^+^ and CD4^+^IL-13^+^ cells in groups of C57BL/6 mice treated with IL-33 at the day 0 and day 6 (also treated with MLD-STZ) as shown in Figures 2, 6. These findings were also observed in NOD mice treated with IL-33 (Figure 7).

As Th2 immune response has a protective role in pathogenesis of diabetes (36), it is possible that one of potential mechanisms of attenuation of the disease mediated by IL-33 is directing immune response toward Th2. However, low dose CY sensitivity of IL-33 antidiabetogenic effect suggests that CD4^+^Foxp3^+^ positive cells are the most important regulators in MLD-STZ model in particular ST2^+^ cells of this subset (Figure 5). The activity of Th1 immune response is controlled by the regulatory T lymphocytes (Tregs), which play a key role in maintaining immune homeostasis. Their quantitative and/or qualitative deficiency is often observed in autoimmune and/or inflammatory diseases. IL-33 is an essential cytokine for induction of ILC2. IL-33 promotes and maintains Tregs directly (37) by affecting maturation of antigen presenting cells (13, 14) and by intrinsic activation of ILC2 which affect Tregs via ICOSL-ICOS interaction (37). In this paper we did not evaluate possible contribution of ILC2 which is shown to play a role in metabolic disorders such as obesity and type 2 diabetes but not in T cells mediated autoimmune diseases such as diabetes or EAE which have similar pathogenesis (38, 39). Alteration of antigen presenting cells as previously shown in EAE and MLD-STZ as well as direct effect of IL-33 on maintenance of Foxp3^+^ST2^+^Treg^+^ appear to be explanations of observed preventive and therapeutic effect of IL-33 in our model. However, it remains to be elucidated whether ILC2 may also have importance in Th1 mediated autoimmunity as shown in lymphoid cell activation during immune perturbation metabolic disorders (37, 40, 41) and allergic inflammation (42).

It was shown that ST2^+^ Tregs are expanded through IL-33-driven production of IL-2 by CD11c^+^ DC. IL-33/IL-2 axis in CD11c^+^ DC has the ability to preferentially expand an activated subset of suppressive Foxp3^+^ Treg expressing ST2. The development of Tregs induces the consumption of IL-2 and therefore the inability to develop Th1 subpopulation.

Several studies have shown the immunoregulatory effect of IL-33 on regulatory cells in the mouse model of inflammatory bowel disease (28) and in mice following cardiac transplantation (43). In both of these studies, IL-33 causes upregulation of CD4^+^CD25^high^Foxp3^+^T cells followed by increasing their number, promoting suppressive activity, as well as ST2 surface expression (28, 43). In addition, two recently published papers have confirmed that IL-33 has the ability to induce regulatory phenotype promoting the expansion of ST2^+^Tregs (43, 44). *In vitro* treatment of mononuclear cells with IL-33 increases the number of CD4^+^CD25^high^Foxp3^+^ cells expressing the ST2 molecule (45).

Our study also showed that the exogenous IL-33 leads to the expansion of CD11c^+^IL-2^+^ cells (Figures 3M,N) and increased number of CD4^+^Foxp3^+^ST2^+^ (Figures 3C,D) immunoregulatory cells that suppress the development of diabetes in mice treated with IL-33 during the induction of the disease. The importance of CD11c^+^CD11b^+^ dendritic cells was indicated previously. It was shown that this DC subset has tolerogenic characteristics and that could be used for immunotherapy of autoimmune diseases (46, 47).

Thus, we showed that exogenous IL-33 attenuates MLD-STZ diabetes induction. The cellular basis of this prevention appears to be mainly due to increased number of Foxp3^+^ cells in particular those expressing ST2 molecule.

The treatment of IL-33 of 16 weeks old NOD mice in our experiment significantly suppressed the development of mononuclear infiltrates in the pancreatic islets. We showed increased percentage of downregulatory cells in IL-33 treated NOD mice (Figure 7). Whether the mechanism is the same as in MLD-STZ diabetes remains to be elucidated. As it has been shown recently that Type 1 interferon inhibits IL-10 signaling and development of diabetes in NOD mice which is promoted by IL-33, the mechanism may be similar (48).

## Author contributions

SP, BL, IP, NJ, and MM performed experiments. SP, BL, IP, NA, and MM analyzed data. ML, NA, and SP conceived and designed experiments. SP and ML wrote the article.

### Conflict of interest statement

The authors declare that the research was conducted in the absence of any commercial or financial relationships that could be construed as a potential conflict of interest.

## Supplementary material

Figure S1 Gating strategy and representative dot and contour plots for the data presented in Figure 2 and Figure 3. The main findings are indicated in corresponding quadrant.

Figure S2 Gating strategy and representative dot and contour plots for the data presented in Figure 4. The main findings are indicated in corresponding quadrant.

Figure S3 Representative dot and contour plots for the data presented in Figure 7. The main findings are indicated in corresponding quadrant.
