# Supplementary figures and images for: IL-33 Prevents MLD-STZ Induction of Diabetes and Attenuate Insulitis in Prediabetic NOD Mice
Source: Front Immunol. 2018 Nov 15;9:2646. doi: 10.3389/fimmu.2018.02646 (PMC6249384; doi:10.3389/fimmu.2018.02646)

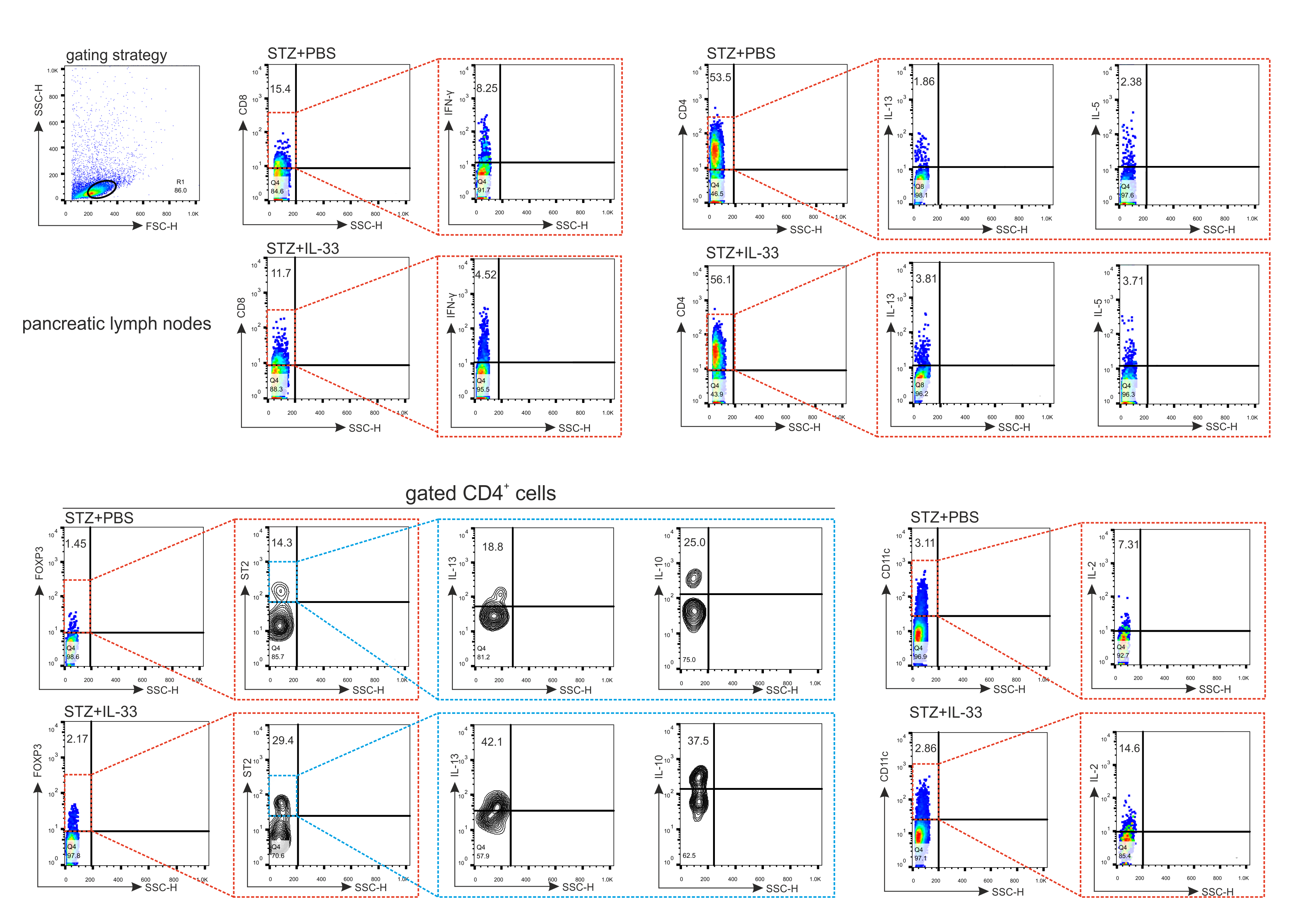

Supplement: Figure S1 — Gating strategy and representative dot and contour plots for the data presented in Figure 2 and Figure 3. The main findings are indicated in corresponding quadrant. [file Image_1.TIF]

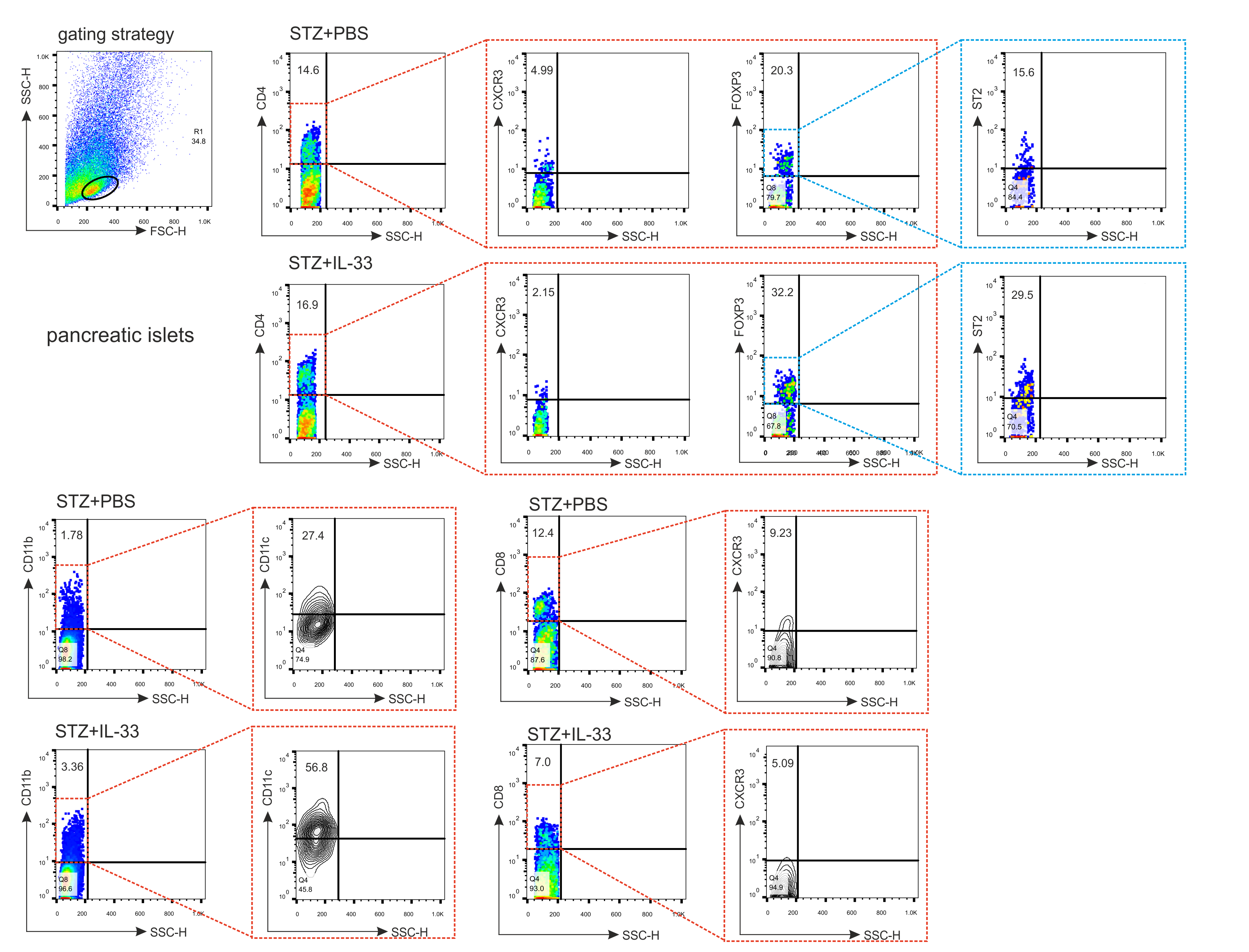

Supplement: Figure S2 — Gating strategy and representative dot and contour plots for the data presented in Figure 4. The main findings are indicated in corresponding quadrant. [file Image_2.TIF]

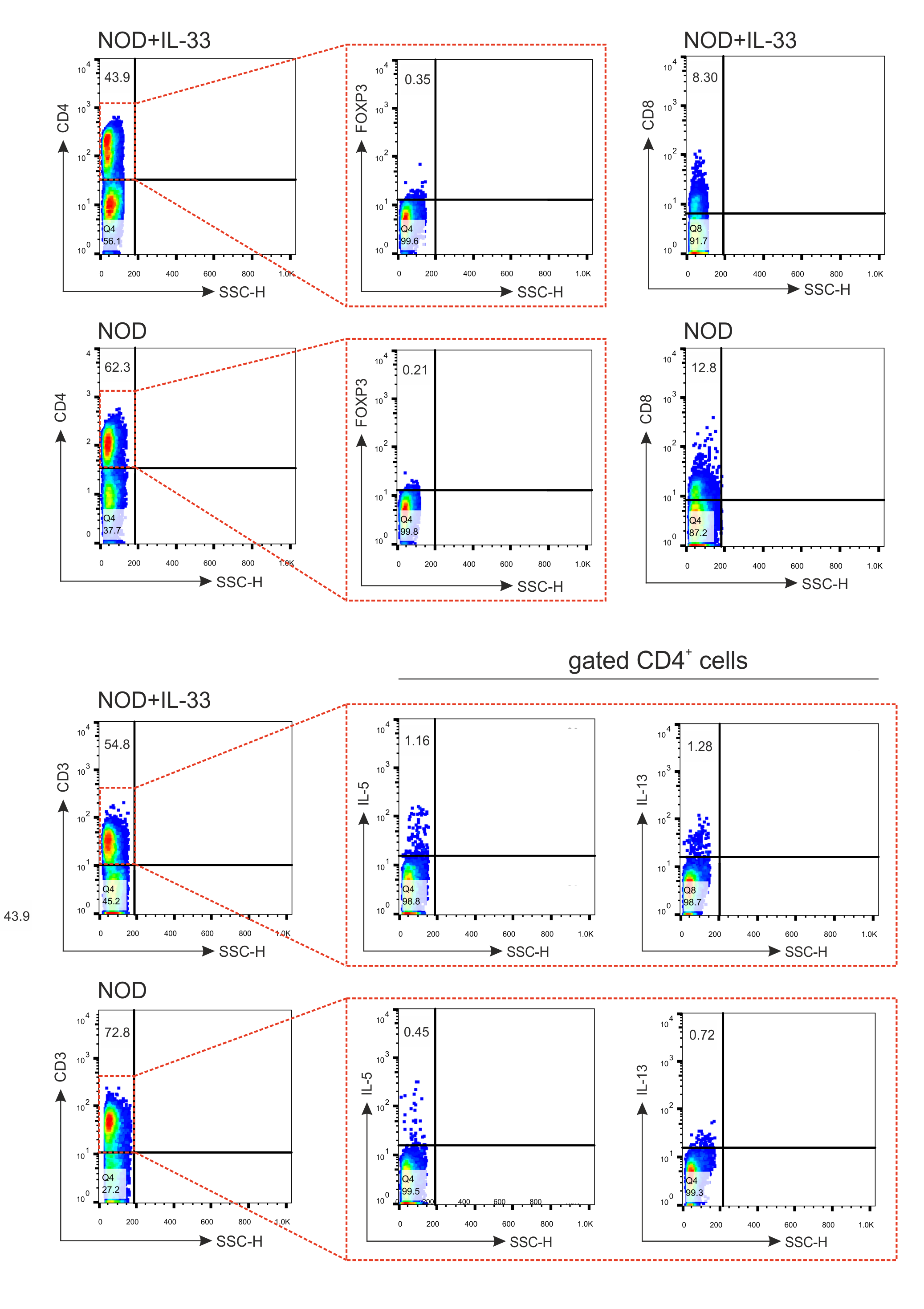

Supplement: Figure S3 — Representative dot and contour plots for the data presented in Figure 7. The main findings are indicated in corresponding quadrant. [file Image_3.TIF]
